# Adhesive Antimicrobial Peptides Containing 3,4-Dihydroxy-L-Phenylalanine Residues for Direct One-Step Surface Coating

**DOI:** 10.3390/ijms222111915

**Published:** 2021-11-03

**Authors:** Young Eun Hwang, Seonghun Im, Hyun Kim, Jung-Hoon Sohn, Byung-Kwan Cho, Ju Hyun Cho, Bong Hyun Sung, Sun Chang Kim

**Affiliations:** 1 Department of Biological Sciences, Korea Advanced Institute of Science and Technology, Daejeon 34141, Korea; s0ag@kaist.ac.kr (Y.E.H.); bcho@kaist.ac.kr (B.-K.C.); 2 Synthetic Biology and Bioengineering Research Center, Korea Research Institute of Bioscience and Biotechnology, Daejeon 34141, Korea; lsh01573@gmail.com (S.I.); sohn4090@kribb.re.kr (J.-H.S.); 3 Division of Applied Life Science (BK21Four), Research Institute of Life Sciences, Gyeongsang National University, Jinju 52828, Korea; hyun.kim@gnu.ac.kr (H.K.); juhyun.cho@gnu.ac.kr (J.H.C.)

**Keywords:** antimicrobial peptide, coating, infection, L-DOPA, in vivo bactericidal activity

## Abstract

Bacterial colonization and transmission via surfaces increase the risk of infection. In this study, we design and employ novel adhesive antimicrobial peptides to prevent bacterial contamination of surfaces. Repeats of 3,4-dihydroxy-L-phenylalanine (DOPA) were added to the C-terminus of NKC, a potent synthetic antimicrobial peptide, and the adhesiveness and antibacterial properties of the resulting peptides are evaluated. The peptide is successfully immobilized on polystyrene, titanium, and polydimethylsiloxane surfaces within 10 min in a one-step coating process with no prior surface functionalization. The antibacterial effectiveness of the NKC-DOPA_5_-coated polystyrene, titanium, and polydimethylsiloxane surfaces is confirmed by complete inhibition of the growth of *Escherichia coli*, *Pseudomonas aeruginosa*, and *Staphylococcus aureus* within 2 h. The stability of the peptide coated on the substrate surface is maintained for 84 days, as confirmed by its bactericidal activity. Additionally, the NKC-DOPA_5_-coated polystyrene, titanium, and polydimethylsiloxane surfaces show no cytotoxicity toward the human keratinocyte cell line HaCaT. The antimicrobial properties of the peptide-coated surfaces are confirmed in a subcutaneous implantation animal model. The adhesive antimicrobial peptide developed in this study exhibits potential as an antimicrobial surface-coating agent for efficiently killing a broad spectrum of bacteria on contact.

## 1. Introduction

Antimicrobial peptides (AMPs) exert direct and rapid antimicrobial activities against various microbial species, including multidrug-resistant bacteria, fungi, and viruses. AMPs operate by destroying the cell membrane leading to cell lysis or by penetrating the cell membrane to act on intracellular targets [1]. Due to the electrostatic interactions between the cationic AMPs and the negative charges on the microbial surfaces, it is difficult for microbes to preserve the physical and functional integrity of the cell membrane against the membrane-disrupting activity of AMPs [2]. Thus, AMPs exhibit a negligible propensity to trigger resistance development. In addition, AMPs display high selectivity toward bacterial cells over eukaryotic cells because mammalian cell membranes consist of zwitterionic phospholipids and cholesterol, which have a neutral net charge [3]. Therefore, AMPs have attracted attention as promising alternatives to conventional antibiotics [4].

Because of these advantages, several studies have focused on using AMPs to prevent and treat microbial infections. Antimicrobial coatings have been developed to prevent contact-transmitted infection, which is among the main routes of infection [5]. When bacteria come into close contact with antimicrobial-coated surfaces, they are killed prior to the proliferation and biofilm formation stage [6,7]. Various coating methods have been used to physically or chemically bind AMPs to the surface of materials, such as implants, catheters, and contact lenses. These methods include layer-by-layer assembly [8], adsorption [9], covalent bonding [10,11], and polymer brushing [12,13]. Although these methods facilitate efficient immobilization of peptides, most of these approaches are suitable only for specific surfaces. In addition, both antimicrobials and surfaces require a multistep coating process, such as surface modification, functionalization, and AMP conjugation [12], which is generally time-consuming, requires harsh chemicals, and involves complicated treatment strategies [14,15].

Bio-inspired peptide coatings have been generated from mussel foot proteins to enable simple and universal binding to the surfaces of various materials [16]. The catechol-containing amino acid 3,4-dihydroxy-L-phenylalanine (DOPA) is the major component responsible for the unique adhesive properties of mussel foot proteins [7]. Catechol strongly adheres to various organic or inorganic surfaces via different types of interactions, including hydrogen bonds, hydrophobic interactions, cation–π interactions, π–π interactions, and covalent crosslinks [17]. The exact adhesion mechanism has not yet been revealed; however, it is believed to be associated with the oxidation of a catechol to a quinone and subsequent reactions [17]. Motivated by the outstanding adhesion ability of DOPA-rich mussel foot proteins, catechol derivatives have been extensively studied for the engineering of surface-coating materials [18,19]. Most studies of catechol-based coatings have been conducted using polydopamine (PD) [20], because its simple two-step coating process can be easily performed for virtually all surface types. The first step involves creating a stable PD coating layer, and the second is anchoring the AMPs on PD [21]. Since DOPA contains additional carboxyl groups that can be directly incorporated into the peptide sequence compared to dopamine, recently developed DOPA-containing peptides can coat the surface without any prior treatment [9]. Although the aforementioned study highlighted the possibility of generating an adhesive anti-fouling peptide by combining DOPA, antimicrobial, and anti-fouling residues, its adhesion and antibacterial activities were not satisfactory. Thus, the development of chimera peptides with strong antimicrobial and binding activities to various surfaces without preprocessing is desirable.

Herein, novel adhesive AMPs were synthesized by combining potent AMPs and varying numbers of DOPA residues, and their feasibility as antimicrobial coatings was demonstrated. Their antimicrobial activity, surface-binding property, cytotoxicity, and long-term stability were analyzed in vitro. Additionally, the effectiveness of the AMP-coated implants in resisting bacterial infection in vivo was confirmed using a subcutaneous implantation animal model. The adhesive peptides developed in this study can be directly employed as universal coating agents by a simple dip-coating process and can be used to efficiently kill bacteria upon contact.

## 2. Results and Discussion

### 2.1. Design of Adhesive AMPs

**Figure 1:**
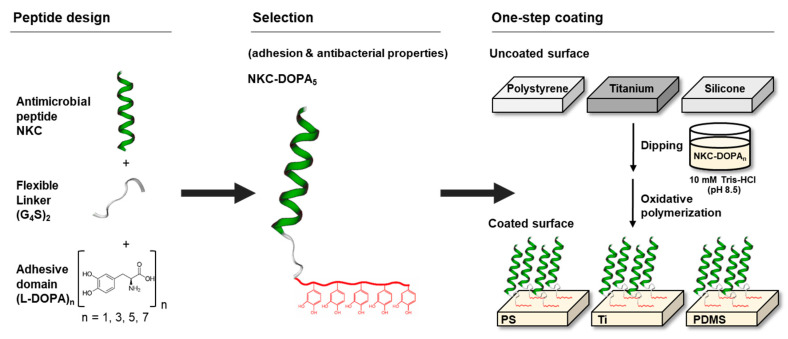
Schematic illustration of the antimicrobial peptide (AMP) design and one-step process for AMP coating.

NKC, a potent AMP, and DOPA, an amino acid that contributes to the adhesion of mussel foot proteins, were combined to develop a peptide capable of attachment to various surfaces without special treatment (Figure 1). The NKC peptide, which has a cationic charge (+7) and an amphipathic α-helical structure, exhibits potent antimicrobial activities against gram-negative and gram-positive bacteria and even fungi, with no cytotoxicity towards human cells [22,23]. A glycine–serine linker (GGGGSGGGGS) was added between NKC and DOPA to provide sufficient flexibility for NKC to bind to bacterial cell membranes and kill bacteria via pore formation [24]. NKC-DOPA derivatives with 1, 3, 5, and 7 DOPA moieties (namely, NKC-DOPA_1_, NKC-DOPA_3_, NKC-DOPA_5_, and NKC-DOPA_7_) were synthesized to identify optimal adhesive peptides. Table 1 summarizes the characteristics of the peptides.

The antimicrobial activity of the synthesized peptides against *E. coli* was determined by measuring the MIC, the lowest concentration of the antimicrobial agent with no visible growth of microorganisms. The NKC peptide had an MIC value of 1 μM, but the MIC values of all peptides fused with the (G_4_S)_2_ flexible linker, with and without L-DOPA, increased to 16 μM. Thus, the antimicrobial activity seemingly decreased with the addition of the flexible linker to NKC. Nevertheless, the antibacterial activity remained sufficient for the peptides to be used as antibacterial coating agents.

### 2.2. Adhesion Capacities of the Designed Peptides

**Figure 2:**
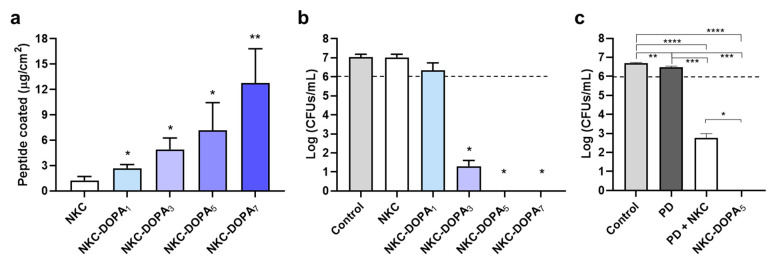
Surface adhesion and antibacterial activities of attached peptides with different numbers of 3,4-dihydroxy-L-phenylalanine (DOPA) residues. Each peptide solution was added to 24-well polystyrene (PS) plates and coated for 10 min. The amounts of adhered peptides were estimated using a bicinchoninic acid assay (**a**). To the coated wells, 10^6^ cells/mL of *E. coli* was added. After 2 h, the surviving cells were plated for colony counting (**b**). Uncoated wells were used as negative controls. The antimicrobial activity of the NKC-DOPA_5_ peptide was compared with that of the NKC peptide, whose surface attachment was mediated by polydopamine (PD) (**c**). The dotted line represents the cell concentration in the initial inoculum. Error bars represent the standard deviation. * *p* < 0.05, ** *p* < 0.01, *** *p* < 0.001, and **** *p* < 0.0001.

The degree of adhesion of NKC-DOPA_n_, depending on the number of DOPA residues, was measured on 24-well PS plates. As the number of DOPA residues increased from 1 to 3, 5, and 7, the masses of the adsorbed peptide proportionally increased, with coverages of 2.7, 4.9, 7.2, and 12.8 μg/cm^2^, respectively (Figure 2a). The binding forces between DOPA and various surfaces are 60–90 pN at a pulling speed of 1000 nm/s [19]; hence, the 2.8-kDa peptide NKC-L, with only one DOPA residue, sufficiently adhered to the surface. However, larger numbers of peptides attached as the number of DOPA residues increased. The antimicrobial activities of PS-attached AMPs with different numbers of DOPA residues against *E. coli* are shown in Figure 2b. The surface coated with NKC-DOPA_1_ hardly inhibited bacterial growth, whereas the NKC-DOPA_3_-coated surface showed a 5.7 log reduction in the cell count. No bacterial growth was observed on surfaces coated with NKC-DOPA_5_ and NKC-DOPA_7_. Surviving bacteria can develop biofilms, increasing their resistance to antimicrobials, and thus complete eradication is necessary. Therefore, in subsequent experiments, NKC-DOPA_5_ was used because a larger number of DOPA residues in the peptide would further increase the cost of synthesis.

To compare antimicrobial activities of a directly and indirectly coated peptide, NKC peptide solution was added to the PD-coated surface of a PS plate. The amount of peptide adhered to the PD-coated surface was 13.6 μg/cm^2^. The directly attached peptides completely inhibited bacterial cell growth. However, the NKC peptides attached to PD reduced the cell count by approximately 4.2 log (Figure 2c), although the amount of indirectly coated peptides was higher than that of directly coated peptides via the fused DOPA residues. This was possibly attributed to the ability of some peptide molecules to freely exert their antibacterial activity, whereas some peptides may have been inhibited by their attachment to PD. Although peptides can easily be immobilized on PD-coated surfaces via Schiff base-mediated biomodification, Schiff base formation could weaken the functionality of biologically active molecules by blocking peptide amino groups [25].

### 2.3. Characterization of AMP-Coated Surfaces

**Figure 3:**
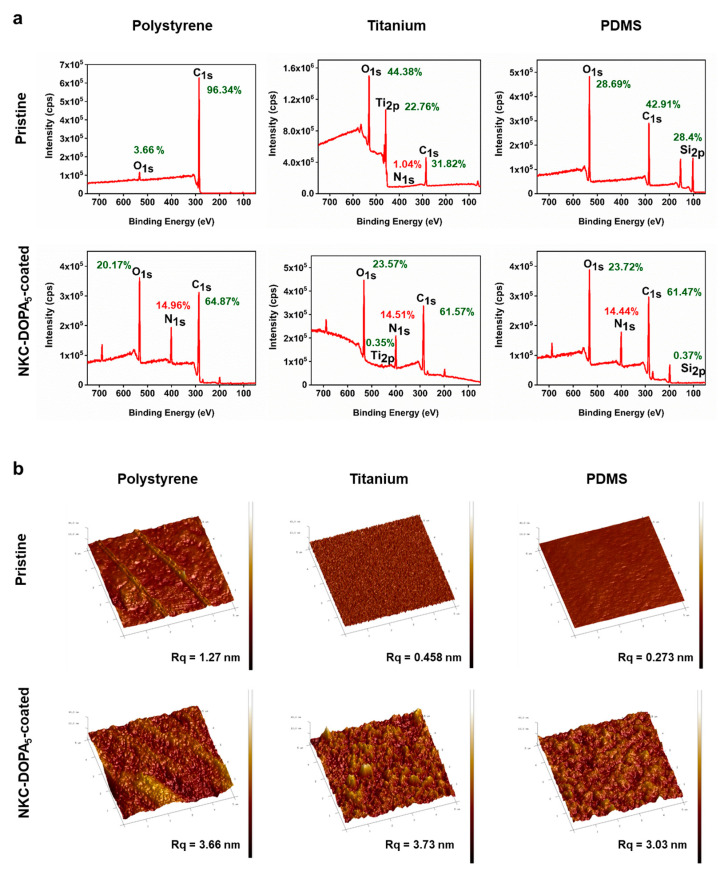
Characteristics of NKC-DOPA_5_-coated surfaces. (**a**) X-ray photoelectron spectroscopy survey spectra of pristine and NKC-DOPA5-coated PS, Ti, and PDMS. (**b**) Atomic force microscopy images of pristine and NKC-DOPA5-coated PS, Ti, and PDMS.

The atomic percentages and the elemental composition of AMP-coated surfaces of PS, Ti, and PDMS were analyzed by XPS [26]. Figure 3a shows the XPS spectra for the pristine and NKC-DOPA_5_-coated surfaces. It was observed that N_1s_ is a good elemental marker because the pristine surfaces contained no N atoms that are had by peptides [15]. The XPS spectrum of pristine PS showed two dominant peaks: C_1s_ (285 eV) and O_1s_ (530 eV). As expected, the NKC-DOPA_5_ (C_168_H_268_N_42_O_48_S_1_) coating on PS resulted in higher percentages of elemental N (0–14.96 atomic percentage) at a binding energy of 400 eV in the spectra. The spectrum of pristine Ti showed three dominant peaks: C_1s_ (285 eV), O_1s_ (530 eV), and Ti_2p_ (454 eV). When NKC-DOPA_5_ was coated onto Ti, a distinct N_1s_ peak appeared at an atomic percentage as high as 14.51. The appearance of N_1s_ and the disappearance of Ti_2p_ in the survey spectrum of NKC-DOPA_5_-coated Ti indicated the successful deposition of NKC-DOPA_5_ on Ti. Consistent findings were obtained for the PDMS surface. The spectrum of NKC-DOPA_5_-coated PDMS showed a remarkable increase in the N_1s_ signal and decrease in the Si_2p_ signal compared to that in the spectrum of pristine PDMS. These results confirm that NKC-DOPA_5_ was successfully coated onto PS, Ti, and PDMS, which was also supported by the increased N/C ratio in the elemental composition of the NKC-DOPA_5_-coated surface. The NKC-DOPA_5_-coated substrates yielded N/C ratios of 0.231–0.236, which were similar to the theoretical N/C ratio of 0.25 for NKC-DOPA_5_, thus indicating that the peptide was almost saturated on the surfaces.

The morphologies of the pristine and NKC-DOPA_5_-coated surfaces were characterized by atomic force microscopy, as shown in Figure 3b. A homogeneous distribution of NKC-DOPA_5_ was observed on the PS, Ti, and PDMS surfaces, which showed increased roughness compared to pristine surfaces. The unmodified substrates had a smooth morphology, with surface roughness values of 1.27, 0.458, and 0.273 nm for the PS, Ti, and PDMS surfaces, respectively. Coating with NKC-DOPA_5_ increased the surface roughness to 3.66, 3.73, and 3.03 nm for the PS, Ti, and PDMS surfaces, respectively. No uncoated regions were observed, and the peptide coverage was uniform, demonstrating successful anchoring of the peptide molecules onto the substrates without the need for prior surface activation and functionalization.

### 2.4. Antimicrobial and Antifouling Efficacies of NKC-DOPA_5_

**Figure 4:**
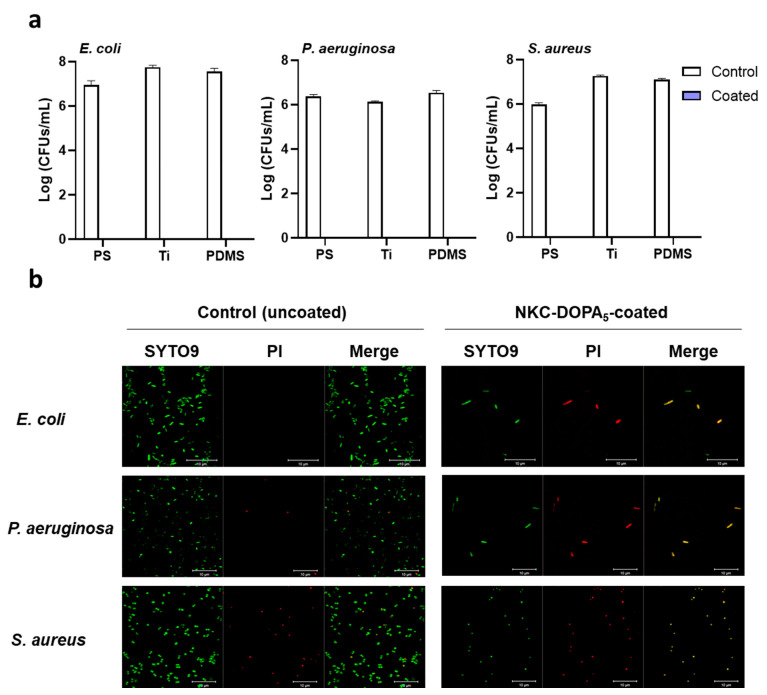
Antibacterial activities of NKC-DOPA_5_ coating. (**a**) Surface antimicrobial activities of NKC-DOPA_5_ coating against *E. coli*, *P. aeruginosa*, and *S. aureus*. (**b**) Bacterial biofilm formation on pristine and NKC-DOPA_5_-coated surfaces after their immersion in bacterial suspensions for 24 h.

The antimicrobial effects of the NKC-DOPA_5_ coating were evaluated against the gram-negative bacteria *E. coli* and *p. aeruginosa* and gram-positive bacterium *S. aureus*, which are frequently associated with human infections. To simulate bacterial contamination, high concentrations of the bacteria were added in small volumes to the test surfaces, as described previously [27]. The AMP-coated and uncoated substrates (1 cm × 1 cm) were placed into 24-well plates, and the bacterial suspensions were distributed over the substrate surfaces. In all cases, the numbers of viable bacterial cells were reduced to zero after 2 h of incubation on the NKC-DOPA_5_-coated surfaces (Figure 4a). These results demonstrate that NKC-DOPA_5_ coatings can be applied to various substrates possessing strong antimicrobial effects.

The attachment of bacteria to solid surfaces initiates biofilm formation, which plays an essential role in the pathogenesis of infections. Because bacteria can thrive on surfaces for up to several months, contaminated environmental surfaces act as microbial reservoirs with the potential to transmit pathogens cause infections in healthy individuals [28,29]. To investigate the inhibition of bacterial biofilm formation by the coated peptides, the bacterial cells were observed on NKC-DOPA_5_-coated surfaces by confocal laser-scanning microscopy following live/dead staining. SYTO9 permeates bacterial membranes and stains the cells as green, whereas PI penetrates only dead cells with damaged membranes and stains the cells as red [30]. The microscopy images in Figure 4b show that most bacterial cells attached to the control (uncoated) surfaces were stained with SYTO9, suggesting that these cells had intact membranes. In contrast, there were few bacterial cells on the NKC-DOPA_5_-coated surfaces and all cells were stained with PI, indicating that their membranes were compromised and that the bacteria could be dead. Based on these results, the peptide coating remarkably inhibited biofilm formation by both gram-negative (*E. coli* and *P. aeruginosa*) and gram-positive (*S. aureus*) bacteria, which can adhere to and proliferate on uncoated surfaces.

### 2.5. Characteristics of the Coated Peptide

**Figure 5:**
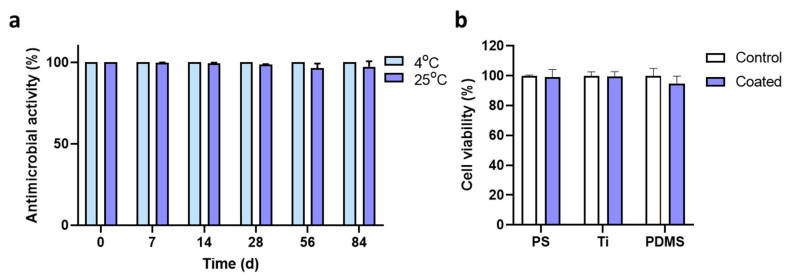
Characteristics of NKC-DOPA_5_ coated on the substrate surface. (**a**) NKC-DOPA_5_-coated 24-well polystyrene plates were stored at 4 °C and 25 °C for 84 days, and the antibacterial activity against *E. coli* was measured every week. (**b**) Viability of HaCaT cells incubated with the uncoated or NKC-DOPA_5_-coated surface was measured and expressed as a percentage relative to that of the uncoated control.

Antibacterial activity was measured following storage of PS plates with immobilized NKC-DOPA_5_ at 4 °C and 25 °C for 84 days to analyze the stability of the immobilized peptide. During the storage period at 4°C, there was no change in the antimicrobial activity of NKC-DOPA_5_ toward *E. coli*. When the coated PS was stored at 25 °C, the antimicrobial activities were 99.75 ± 0.16% and 99.17 ± 3.25% after 7 and 84 days, respectively (Figure 5a). These results suggest that the NKC-DOPA_5_ coating is highly stable under suitable storage conditions and maintains sufficient biocidal capability for approximately 3 months.

The potential cytotoxicity of the NKC-DOPA_5_-coated substrates against HaCaT cells was determined in an MTT assay. The coated substrates placed on HaCaT cells hardly affect cell viability, as the viability was >90% for all tested substrates (Figure 5b). These results were expected, as NKC has been reported as being non-toxic toward mammalian cells [22,24].

### 2.6. In Vivo Antimicrobial Activity of NKC-DOPA_5_

**Figure 6:**
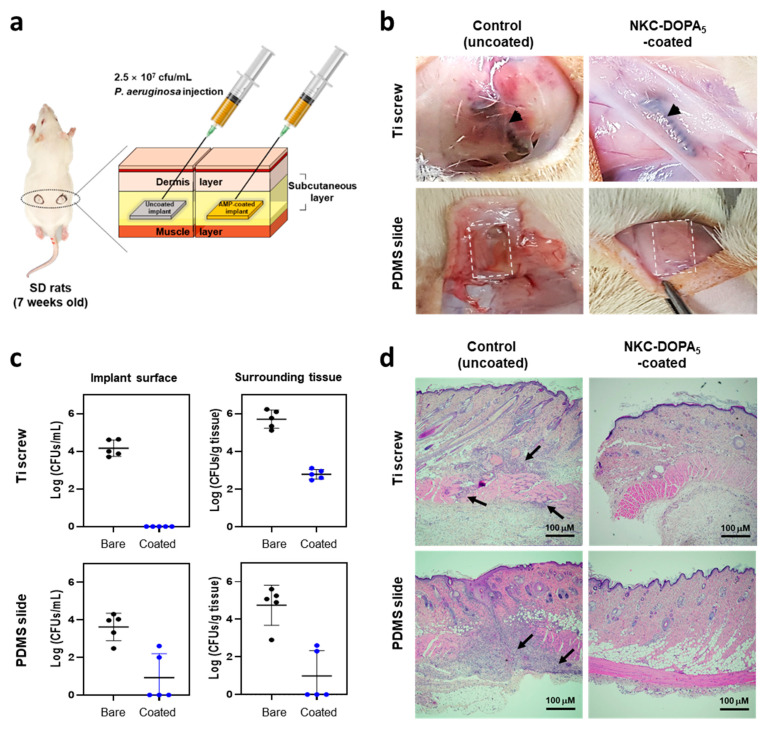
In vivo antimicrobial activity of NKC-DOPA_5_ coated on implant surfaces. (**a**) Schematic illustration of the in vivo infection model. Uncoated and NKC-DOPA_5_-coated implants were placed in the subcutaneous tissue on the back of Sprague–Dawley (SD) rats. *Pseudomonas aeruginosa* (2.5 × 10^8^ colony-forming unit (CFU)/mL, 100 µL) was injected at the implant site. (**b**) Images of excisional wounds at 5 days after surgery in the control group, implanted with an uncoated Ti screw or polydimethylsiloxane (PDMS), and in the sample group, implanted with an NKC-DOPA_5_-coated Ti screw or PDMS. Black arrows and white dotted frames indicate the positions of the Ti screw and PDMS slide, respectively. (**c**) CFU counts of *P. aeruginosa* on each implant and surrounding tissues in the uncoated and AMP-coated groups. (**d**) Hematoxylin and eosin-stained sections of tissues surrounding the uncoated and NKC-DOPA_5_-coated implants on day 5 (magnification: 40×). Arrows indicate inflammatory cell infiltration.

The performance of NKC-DOPA_5_-coated implants in vivo was evaluated (Figure 6a), as described previously, using a rat subcutaneous implant model [31]. The implants and surrounding tissues were harvested from the rats at 5 days post-inoculation of *P. aeruginosa*, and the location of implants was unchanged with respect to the skin marks. The implant sites of the uncoated Ti screws and PDMS showed inflammation (Figure 6b). In contrast, tissues around the NKC-DOPA_5_-coated implants were integrated without apparent inflammation. The bacterial CFU counts on each implant and in the surrounding tissues are presented in Figure 6c. In the Ti-uncoated (control) group, all five implants exhibited a high microbial load of up to 4.2 log CFU/mL per implant on average. In contrast, in the NKC-DOPA_5_-coated Ti-implanted group, no microorganisms were observed on any of the five implants. All implants showed an average microbial load as high as 3.6 log CFU/mL per implant in the PDMS control group, whereas in the NKC-DOPA_5_-coated PDMS-implanted group, microorganisms were absent from three of the five implants. The other two implants showed microbial loads of 2.0−2.6 log CFU/mL and an average bacterial count of 0.92 log CFU/mL per implant [32]. A 3 log CFU/g difference in the numbers of bacteria from the tissues surrounding the Ti implants was observed between the pristine (5.7 log CFU/g) and NKC-DOPA_5_-coated (2.7 log CFU/g) Ti implants. For the PDMS implants, the NKC-DOPA_5_ coating decreased the number of bacteria recovered from the surrounding tissues by approximately 3.8 log CFU/g compared with that in the pristine PDMS group, with median numbers of 0.9 and 4.7 log CFU/g, respectively. No microorganisms were observed on three of the five NKC-DOPA_5_-coated PDMS implants. It has been reported that a tissue bacterial load of more than 5 log CFU/g causes acute or chronic wound infection [32]; therefore, the NKC-DOPA_5_ coating may provide sufficient protection by killing most of the microbes. In Figure 6d, hematoxylin and eosin staining of the surrounding tissues showed that bacterial injection exacerbated inflammation in the area surrounding the uncoated Ti and PDMS implants. High levels of inflammatory cell infiltration were observed in the tissues surrounding the uncoated Ti and PDMS implants, indicating severe infection at these sites. Additionally, the numbers of inflammatory cells detected by histological examination of the tissues surrounding the NKC-DOPA_5_-coated implants were not significant, indicating that the NKC-DOPA_5_-coated samples did not cause high levels of inflammation. These in vivo results validate the effectiveness of NKC-DOPA_5_ coating in preventing implant-associated infections caused by *P. aeruginosa* in animals.

Numerous studies have been performed to develop coating agents using AMPs. Several factors, such as the economic feasibility of production as well as the safety and stability of the peptide as an antibacterial coating agent, must be considered when developing these AMPs. As the adhesive AMP developed in this study contains the amino acid DOPA, it can be produced using a biological expression system. This peptide may be produced in a DOPA-fused form in genetically engineered amberless *E. coli* strains [33,34] or in a tyrosine-containing form to be replaced with DOPA by tyrosinase. The development of a mass production method for AMPs will enable economically efficient synthesis of antimicrobial coating agents, which will help prevent the spread of infectious diseases.

## 3. Materials and Methods

### 3.1. Peptides, Strains, Reagents, and Substrates

The potent AMP NKC [22,23] and adhesive peptide candidates, designed based on NKC, were chemically synthesized via solid-phase peptide synthesis by AnyGen Co., Ltd. (Gwangju, Korea) at a purity of > 90%. These peptides were further purified using reverse-phase high-performance liquid chromatography (Shimadzu 20A gradient system; Shimadzu, Kyoto, Japan). Data were collected using an SPD-20A detector at a wavelength of 230 nm. Chromatographic separation was performed using a 1%/min linear gradient of buffer B [0.1% trifluoroacetic acid in acetonitrile] in buffer A (0.1% trifluoroacetic acid in H_2_O) over a period of 40 min at flow rates of 1 and 8 mL/min using Shimadzu C_18_ analytical (5 μm, 0.46 cm × 25 cm) and preparative (10 μm, 2.5 cm × 25 cm) columns. The identity of the peptides was confirmed by mass spectrometry. All lyophilized peptides were stored at −20°C. Before use, the peptides were dissolved in 10 mM Tris-HCl (pH 8.5) at a stock concentration of 2 mM and freshly diluted for subsequent experiments.

*Staphylococcus aureus* ATCC 25923, *Escherichia coli* ATCC 27325, *Pseudomonas aeruginosa* PAO1, and the human keratinocyte cell line HaCaT were acquired from the American Type Culture Collection (Manassas, VA, USA). Bacterial cells were cultured at 37 °C in lysogeny broth (LB; 10 g of tryptone, 5 g of yeast extract, and 5 g of NaCl per liter). Soybean casein digest broth containing lecithin and polyoxyethylene sorbitan monooleate (SCDLP) broth, which is used to recover cells for measuring antibacterial activity, was prepared by dissolving 17 g of casein peptone, 3 g of soybean peptone, 5 g of sodium chloride, 2.5 g of disodium hydrogen phosphate, 2.5 g of glucose, and 1 g of lecithin in 1000 mL of deionized water. The SCDLP medium was thoroughly mixed, and then 7 g of a nonionic surfactant (polyoxyethylene sorbitan monooleate) was added. The pH was adjusted to 6.8–7.2 (at 25 °C) with hydrochloric acid, after which the medium was sterilized by autoclaving.

Unless otherwise specified, all reagents were purchased from Sigma–Aldrich (St. Louis, MO, USA). Polystyrene (PS) 24-well plates were purchased from Corning Inc. (Corning, NY, USA) and cut into 1 cm × 1 cm squares to prepare the PS surfaces. To prepare the titanium (Ti) surfaces, Si wafers with a diameter of 2 inches were coated with a 100-nm Ti layer by electron beam evaporation at a rate of 1 Å/s and diced into 1 cm × 1 cm pieces. Polydimethylsiloxane (PDMS) silicone rubber was prepared using the Sylgard 184 Silicone Elastomer Kit (Dow Corning, Midland, MI, USA) according to the manufacturer’s instructions. Briefly, the base and curing agents from the kit were mixed thoroughly at a 10:1 (*w*/*w*) ratio, cast into a Petri dish, and stored at 25 °C for 72 h. After curing, the PDMS was cut into 1 cm × 1 cm pieces.

### 3.2. Minimum Inhibitory Concentration (MIC) Assay

The MICs of the designed peptides were measured against the gram-negative bacterium *E. coli* ATCC 27325 according to the general guidelines of the Clinical and Laboratory Standards Institute, with some modifications based on a study of cationic AMPs [35]. Briefly, a single bacterial colony was cultured in Mueller−Hinton broth (MHB; Sigma–Aldrich) until the mid-log phase (optical density of 0.4–0.6 at 600 nm). The bacterial suspension was diluted with fresh MHB to a concentration of 10^6^ colony-forming units (CFU)/mL. Next, two-fold serial dilutions (128 to 1 μM) of the peptides were prepared in MHB and mixed (1:1) with the diluted bacterial culture, resulting in a final peptide concentration ranging from 64 to 0.5 μM and final bacteria concentration of 5 × 10^5^ cells/mL. The mixtures were placed into 96-well polypropylene microtiter plates (Corning), with sterile MHB used as a negative control and MHB inoculated with bacterial cells used as growth control. The plates were incubated for 18 h at 37 °C with shaking at 200 rpm. The MIC values were determined as the lowest concentrations at which no bacterial growth was recorded at 600 nm using a microplate reader (Infinite 200 Pro; Tecan, Männedorf, Switzerland).

### 3.3. AMP Coating

To coat the designed peptides on a surface, 200 μL of freshly prepared 200 μM peptide solution (10 mM Tris-HCl, pH 8.5) was added into a 24-well PS plate and incubated for 10 min at 37 °C. After coating, the plates were rinsed with deionized water and air-dried. The PS, Ti, and PDMS pieces (1 cm × 1 cm) were coated in the same manner. For the PD-mediated AMP coating, a layer of PD was first coated on a 24-well PS plate by dip coating, as previously described [21], with minor modifications. Each PS well was treated with 2 mg/mL dopamine solution (10 mM Tris-HCl, pH 8.5) and incubated for approximately 1 h at 37 °C. The PD-coated wells were washed twice with deionized water to remove excess dopamine. To attach the NKC peptide to a PD-coated 24-well PS plate, NKC peptide solution (200 μM) was dispensed into each well, and the plate was incubated at 37 °C for 24 h. The wells with the immobilized peptide were then washed twice with deionized water.

### 3.4. Quantification of Immobilized Peptides

The amounts of the peptides immobilized on the PS surface in a 24-well plate were determined by quantifying the loaded and unattached peptides retrieved immediately after coating, as described previously [36,37]. The peptide concentration was evaluated using a micro bicinchoninic acid assay kit (Thermo Fisher Scientific, Waltham, MA, USA), with bovine serum albumin used as the standard. In a 96-well plate (Corning), 150 μL of each peptide sample was mixed with 150 μL of working bicinchoninic acid solution. The plate was incubated at 37 °C for 2 h, after which the absorbance at 562 nm was measured with a microplate reader (Tecan Infinite 200 Pro) [38]. Uncoated wells were used as negative controls.

### 3.5. Surface Characterization

The surface chemical composition was characterized by X-ray photoelectron spectroscopy (XPS; Thermo K-Alpha XPS, Thermo Fisher Scientific). Monochromatic Al Kα X-rays (hm = 1486.7 eV) were used, and the analysis chamber was evacuated to a pressure of 1 × 10^−9^ mbar. All spectra were acquired with a charge neutralization flood gun and measured at an electron take-off angle of 30° with respect to the surface plane. Survey spectra and high-resolution spectra of C, N, O, Ti, and Si were obtained to identify the surface chemical components and for elemental ratio comparisons [39]. Changes in surface roughness and morphology of uncoated and AMP-coated substrates were analyzed by atomic force microscopy (AFM; INNOVA-LABRAM HR800, Bruker, Billerica, MA, USA). A high-resonance frequency Si cantilever was used, and images were collected in non-contact tapping mode with a scanning area of 5 nm × 5 nm. Images of two independently coated surfaces were collected for each sample.

### 3.6. Antibacterial Activity of Peptide-Coated Surfaces

The bactericidal activities of the AMP-coated surfaces were evaluated following the International Organization for Standardization 22196 standard with slight modifications. Overnight bacterial suspensions were subcultured in fresh LB until the mid-log phase and diluted to 10^6^ cells/mL with 500-fold diluted LB. The AMP-coated and uncoated substrates (1 cm × 1 cm) were placed in 24-well plates, and 10 μL of the bacterial suspension was distributed over the substrate surface. The substrates were covered with a piece of polyethylene film (1 cm × 1 cm) and incubated for 2 h (37 °C, relative humidity: >90%). Subsequently, SCDLP broth (990 μL) was added, and viable cells were retrieved and serially diluted. Aliquots (100 μL) of the bacterial suspension were withdrawn and plated for colony counting.

### 3.7. Bacterial Adhesion Assay

The viability of bacterial cells adhered to the substrate surface was evaluated using the Live/Dead BacLight Bacterial Viability Kit (Thermo Fisher Scientific) according to the manufacturer’s instructions. Briefly, 200 μL of bacterial suspensions adjusted to 10^7^ cells/mL were incubated on uncoated or peptide-coated chambered cover glasses (Thermo Fisher Scientific) at 37 °C for 24 h. After 24 h, planktonic cells were removed, and the surfaces were washed with 0.85% (*w*/*v*) NaCl buffer. Cells attached to the surfaces were stained for 15 min in the dark with BacLight stock solution containing two fluorescent nucleic acid stains, SYTO9 and propidium iodide (PI). After staining, the surfaces were washed with 0.85% NaCl solution, and the stained cells were photographed using confocal laser-scanning microscopy (LSM 5 LIVE DuoScan, Carl Zeiss, Jena, Germany) using a 63× objective. The excitation wavelengths were 488 and 561 nm for SYTO9 and PI, respectively.

### 3.8. Stability Assessment

To evaluate the stability of the surface-coated peptides, antimicrobial activity was determined as described above after incubating the AMP-coated PS plates for 1, 7, 14, 21, 28, 56, and 84 days at 4 °C and 25 °C and with the formula below [40]:

$$\mathrm{Antimicrobial}\;\mathrm{activity}\;\left(\%\right)=\frac{N_{c}-N}{N_{c}}\times 100$$

where *N_c_* and *N* represent the number of colonies after incubation on the control and AMP-coated substrates, respectively.

### 3.9. Cytotoxicity Assay

To evaluate the toxicity of the peptides coated on the substrates towards human skin, the mitochondrial dehydrogenase activity of HaCaT keratinocytes was measured in a modified 3-(4,5-dimethyl-2-thiazolyl)-2,5-diphenyl-2H-tetrazolium bromide (MTT) reduction assay. HaCaT cells were routinely cultured in Dulbecco’s modified Eagle’s medium (DMEM; Lonza, Basel, Switzerland) with 10% fetal bovine serum (Lonza) and 1% penicillin–streptomycin solution (Thermo Fisher Scientific) in humidified 5% CO_2_ at 37 °C. The cultured cells were washed twice with phosphate-buffered saline (PBS; pH 7.4) and detached by trypsinization. The harvested HaCaT cells were diluted to 5 × 10^4^ cells/mL, seeded into 12-well culture plates, and incubated in a humidified incubator with 5% CO_2_. After incubation, the medium was changed to serum-free DMEM, and uncoated or AMP-coated pieces (PS/Ti/PDMS; 1 cm × 1 cm) were placed upside down on the HaCaT cells. After 24 h, the pieces were removed, and MTT solution in 1 mL of DMEM was added to each well. The plate was then incubated at 37 °C for 4 h in a humidified incubator with 5% CO_2_, followed by an addition of 1 mL of Solubilization Solution/Stop Mix (Promega, Madison, WI, USA) per well to dissolve the formazan crystals. The plate was incubated overnight, and the absorbance was measured at 570 nm using a microplate reader (Tecan Infinite 200 Pro). Cells incubated in medium containing 2% Triton X-100 were used as a negative control. Cell viability was expressed as the percentage of surviving cells on the coated substrates relative to those on the uncoated substrates.

### 3.10. In Vivo Antimicrobial Activity in a Rat Subcutaneous Infection Model

In vivo antibacterial activity was evaluated using specific pathogen-free Sprague–Dawley male rats (7 weeks old, weighing 200−220 g). All animal experiments were approved by the Animal Ethics Committee of KNOTUS, Incheon, Korea (approval no. 18-KE-383) and performed following the guidelines for the care and use of laboratory animals of KNOTUS. All rats were housed in individually ventilated cages and acclimated for 7 days before surgery. For implantation, the rats were anesthetized by intramuscular injection of Zoletil^®^ (30 mg/kg; Virbac, Carros, France), and the dorsum was shaved and disinfected with 70% isopropyl alcohol. Approximately 1 cm wide incisions were made parallel to the spine and extended to the subdermal fascia. For the control group, uncoated Ti screws or PDMS slides were implanted into the left flank, whereas AMP-coated implants were inserted into the right flank. The wounds were sutured, and the implant site was marked on the skin. Setting the skin mark as a guide, 100 μL of a *p. aeruginosa* inoculum containing 2.5 × 10^8^ cells/mL was injected subcutaneously. The rats were euthanized by CO_2_ asphyxia at 5 days after the bacterial challenge [31]. The implants were removed using sterile tools and placed in sterile tubes containing 1 mL of PBS. For the detachment of bacteria from the implant materials, the tubes were sonicated for 15 min, and 10-fold serial dilutions of the bacterial solution were plated on LB agar for CFU determination. The tissues surrounding the implants were carefully removed, separately immersed in 1 mL of PBS, and homogenized. Bacteria in the tissues were quantified by serial dilution [41]. For histological examination, the tissues surrounding the implants were formaldehyde-fixed, paraffin-embedded, and then stained with hematoxylin and eosin [26].

### 3.11. Statistical Analysis

All experiments were conducted in triplicate, and the mean ± standard deviation was calculated for all data. The statistical significance of the data was analyzed by unpaired *t*-test. Values of *p* < 0.05 were considered to indicate statistically significant results.

## 4. Conclusions

We developed AMPs containing DOPA moieties as coatings on various material surfaces in a single-step process without intermediate steps. The adhesive AMP NKC-DOPA_5_ was rapidly and successfully immobilized on PS, Ti, and PDMS surfaces. Immobilization of AMPs on material surfaces may provide high local antimicrobial concentrations at target sites and kill approaching microbes. The NKC-DOPA_5_-coated substrates efficiently killed bacteria upon contact, showed good biocompatibility, and maintained sufficient bactericidal capabilities for up to 3 months. Additionally, in vivo experiments showed that peptide-coated PDMS implants and Ti screws could inhibit microbial infection. The adhesive peptide developed in this study is a single molecule produced by chemical synthesis and is easy to apply using a simple dipping method. This surface-binding AMP shows potential as an antimicrobial coating for reducing the risk of bacterial infections, and this substrate-independent coating will facilitate practical applications.

## Figures and Tables

**Table 1:** Adhesive antimicrobial peptides (AMPs) used in this study.

Name	Amino Acid Sequence *	MW (Da)	Reference
NKC	APKAMKLLKKLLKLQKKGI	2148.8	Yang et al. [23]
NKC-L	APKAMKLLKKLLKLQKKGIGGGGSGGGGS	2781.2	This study
NKC-DOPA_1_	APKAMKLLKKLLKLQKKGIGGGGSGGGGSY	2959.6	This study
NKC-DOPA_3_	APKAMKLLKKLLKLQKKGIGGGGSGGGGSYYY	3318.0	This study
NKC-DOPA_5_	APKAMKLLKKLLKLQKKGIGGGGSGGGGSYYYYY	3676.3	This study
NKC-DOPA_7_	APKAMKLLKKLLKLQKKGIGGGGSGGGGSYYYYYYY	4034.7	This study

* Y indicates L-DOPA.

## Data Availability

The datasets generated during and/or analyzed during the current study are available from the corresponding author on reasonable request.

## Abbreviations

AMP: antimicrobial peptide; DOPA, 3,4-dihydroxy-L-phenylalanine; PDMS, polydimethylsiloxane; PD, polydopamine; SCDLP, polyoxyethylene sorbitan monooleate; XPS, X-ray photoelectron spectroscopy; MTT, 3-(4,5-dimethyl-2-thiazolyl)-2,5-diphenyl-2H-tetrazolium bromide; DMEM, Dulbecco’s modified Eagle’s medium.
